# Prognostic Significance of PD-L1 Expression and Standardized Uptake Values in the Primary Lesions of Stage IV Adenocarcinoma Lung Cancer

**DOI:** 10.3389/fmed.2022.895401

**Published:** 2022-05-13

**Authors:** Bui Tien Cong, Pham Cam Phuong, Pham-Van Thai, Vu-Le Thuong, Nguyen Quang Hung, Dong-Thi Hang, Hoang Anh Tuan, Doan Minh Khuy, Pham-Van Tuyen, Nguyen Minh Duc

**Affiliations:** ^1^Department of Nuclear Medicine, Ha Noi Medical University, Hanoi, Vietnam; ^2^Nuclear Medicine and Oncology Center, Bach Mai Hospital, Hanoi, Vietnam; ^3^Department of Examination, Bach Mai Hospital, Hanoi, Vietnam; ^4^Pathology and Cytology Center, Bach Mai Hospital, Hanoi, Vietnam; ^5^Department of Radiology, Pham Ngoc Thach University of Medicine, Ho Chi Minh City, Vietnam

**Keywords:** lung cancer, adenocarcinoma, stage IV, FDG PET/CT, PD-L1, prediction, SUV_max_

## Abstract

**Background:**

This study evaluated the prognostic ability of ^18^F-fluorodeoxyglucose (^18^F-FDG) positron emission tomography (PET)/computed tomography (CT) in patients with stage IV adenocarcinoma lung cancer to detect protein death-ligand 1 (PD-L1) expression levels.

**Methods:**

In total, 86 patients with stage IV adenocarcinoma lung cancer underwent ^18^F-FDG PET/CT imaging and PD-L1 expression evaluation before treatment from February 2019 to November 2020 at Bach Mai Hospital, Hanoi, Vietnam. The assessed patient characteristics in this study included sex, age, smoking status, epidermal growth factor receptor (*EGFR*) mutation, PD-L1 expression level, survival status, tumor, node, and metastasis (TNM) stage, and metastasis locations.

**Results:**

The average age was 62.23 ± 9.51 years, and men and women represented 67.4% and 32.6% of the population, respectively. The *EGFR* mutation rate was 36%. PD-L1 expression was negative (detected in <1% of the tumor) in 40.7% of cases and positive in 59.3% of cases (detected in 1–49% of the tumor in 32.6%; detected in ≥50% of the tumor in 26.7%). The mean maximum standardized uptake value (SUV_max_) was 11.09 ± 3.94. SUV_max_ was significantly higher in PD-L1–positive tumors than in PD-L1–negative tumors (12.24 ± 4.01 and 9.43 ± 3.22, respectively; *p* = 0.001). Receiver operating characteristic curve analysis revealed an area under the curve of SUVmax was 0.681 (95% confidence interval 0.570–0.793, *p* = 0.004). Compared with PD-L1–negative cases, SUV_max_ was significantly different in all PD-L1–positive cases (*p* = 0.001), weakly PD-L1–positive cases (1–49%, *p* = 0.005), and strongly PD-L1–positive cases (≥50%, *p* = 0.003). PD-L1 expression levels were significantly associated with SUV_max_ (*p* = 0.001), tumor size (*p* = 0.022), and *EGFR* mutation status (*p* = 0.045).

**Conclusions:**

SUV_max_ in the primary lesions was able to predict PD-L1 expression and may play a role in predicting PD-L1 immunotherapy efficacy in patients with stage IV lung adenocarcinoma.

## Introduction

Lung cancer is associated with high morbidity and mortality rates. According to GLOBOCAN 2020, lung cancer is the second-most frequent cancer type worldwide (11.4%), accounting for over 2.3 million new cases each year. Lung cancer is estimated to be the leading cause of cancer-related death in both sexes (1) and is the leading cause of cancer-related death in most developed countries (2).

Lung cancer is highly aggressive, rapidly developing, and has poor prognosis, with a 5-year survival rate of only 15% for both sexes (2). However, the majority of individuals are diagnosed at stage III, and late-stage diagnosis (stage IV) is particularly common in developing countries. In recent years, immunotherapy have become increasingly popular treatment options for late stage lung cancer. Testing for programmed death-ligand 1 (PD-L1) expression level should be routinely performed routinely to choose the most suitable treatment approach for patients with non-small cell lung cancer (NSCLC) (3).

Positron emission tomography (PET)/computed tomography (CT) is a nuclear medicine diagnostic tool that enables early detection, provides comprehensive information regarding disease stage, and has prognostic value in patients with NSCLC. Monoclonal antibodies have been developed that target PD-L1, a critical immune system checkpoint. The binding of programmed cell death protein 1 (PD-1) with PD-L1 induces T-lymphocyte depletion or death. The inhibition of this signaling pathway has been shown to increase T cell activity, boost antitumor immunity, and prevent tumor cells from evading host immune responses, representing a viable technique for successful tumor immunotherapy (4).

Numerous studies have been conducted worldwide to ascertain the relationship between the maximum standard uptake value (SUV_max_) and PD-L1 expression. Our published research has not established any relationships between PD-L1 expression levels and SUV_max_ in patients with NSCLC. PD-L1 expression is higher in solid tumors, such as lung cancer, breast cancer, colorectal cancer, and liver cancer, than in other tumor types (5–9).

Limited data is available for late-stage NSCLC, especially stage IV. Additionally, little research has been conducted in Vietnam examining PD-L1 expression in NSCLC patients, and no existing studies in Vietnam have demonstrated a relationship between PET/CT values and PD-L1 expression levels. Therefore, this study evaluated the prognostic significance of ^18^F-fluorodeoxyglucose (^18^F-FDG) PET/CT values to predict different PD-L1 expression levels in patients with stage IV adenocarcinoma lung cancer.

## Materials and Methods

This cross-sectional study was conducted in patients with stage IV adenocarcinoma lung cancer treated at the Nuclear Medicine and Oncology Center and Pathology and Cytology Center, Bach Mai Hospital, from February 2019 to November 2020.

This study was approved by the Ethics Committee of Hanoi Medical University (accession No. NCS02/HMU-IRB), and written informed consent was obtained from all included patients. We included all patients with stage IV NSCLC with an adenocarcinoma histologic subtype treated at our hospital during the study period. Before treatment, patients underwent ^18^F-FDG PET/CT and were tested for PD-L1 expression and *EGFR* mutations. The normal functions of the liver, kidney, and bone marrow were recorded. All patients who agreed to participate in this study provided a complete medical record for study use. Exclusion criteria included lung cancer types and stages other than stage IV adenocarcinoma, no histologic sample from the primary tumor, or lack of *EGFR* mutation or PD-L1 testing. Patients whose primary tumors were not defined on ^18^F-FDG PET/CT were also excluded.

**Figure 1:**
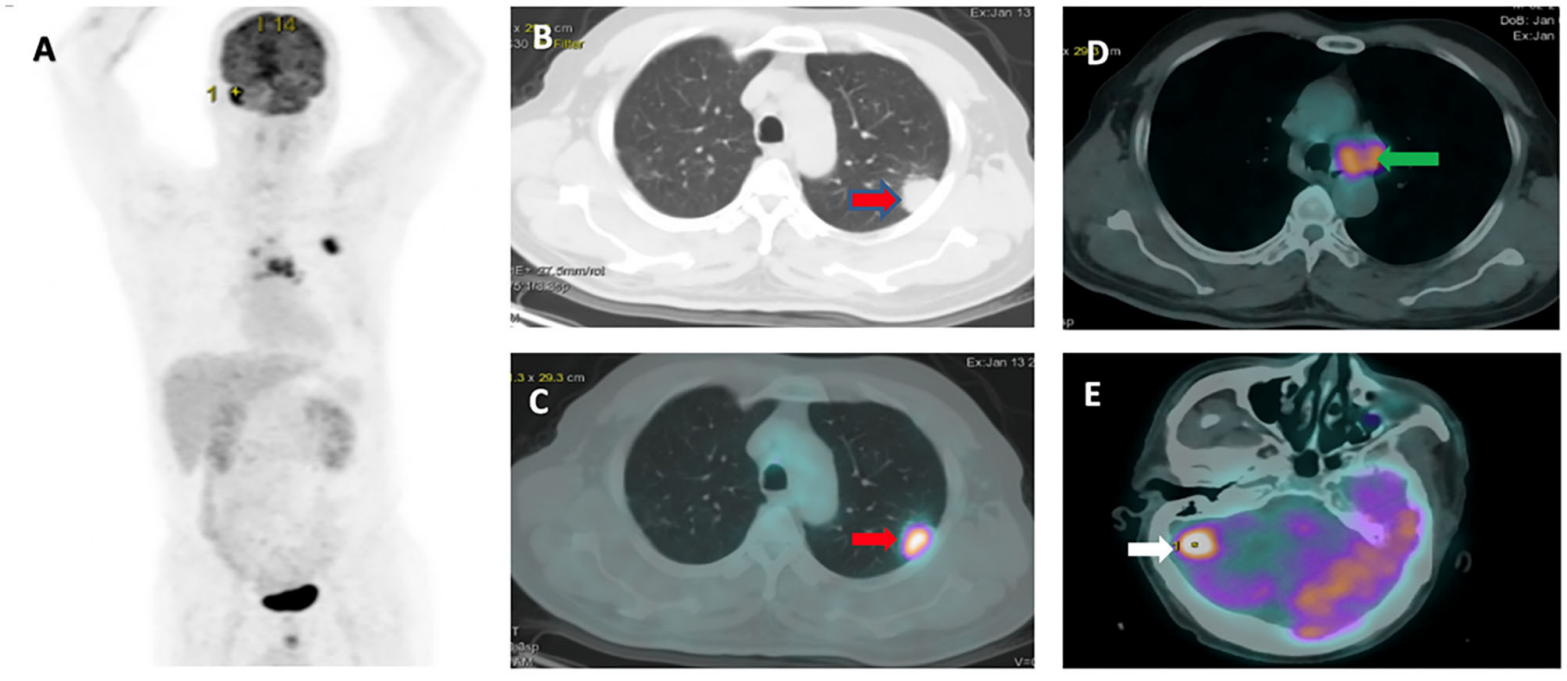
Representative ^18^F-FDG PET/CT images: **(A)** PET image. **(B,C)** Pleural tumor invasion was detected in the upper left lung with SUV_max_ or 10.17 (red arrow). **(D)** Mediastinal node metastasis (green arrow). **(E)** Brain metastasis (white arrow).

The patient characteristics assessed in this study included sex, age, smoking status, *EGFR* mutation, PD-L1 expression level, CEA level, Cyrfra 21-1 level, survival status, tumor, node, and metastasis (TNM) stage, and metastasis locations. Patients were asked to fast for 4 h prior to intravenous administration of 0.15 mCi/kg bodyweight ^18^F-FDG. PET testing was performed 45 min after ^18^F-FDG administration. ^18^F-FDG PET/CT was performed using an ECAT ACCEL (Siemens). Image slices were obtained and analyzed by Syngo Via software from the skull to midthigh vertex. The following variables were assessed using the PET/CT results: primary tumor size, TNM stage, and tumor characteristics. A region of interest (ROI) was manually placed on the lung lesion detected in PET/CT images, and SUV_max_ was obtained. Figure 1 shows one representative patient in our study with TNM staging according to PET/CT.

PD-L1 testing was performed at our institution by an expert pathologist. Samples were obtained from the primary tumor.

### PD-L1 Expression

We followed the instructions provided in the “PD-L1 Immunohistochemistry Testing in Lung Cancer” manual distributed by the International Association for the Study of Lung Cancer (IASLC). Briefly, pathologists counted PD-L1–positive tumor cells, defined as complete circumferential or partial cell membrane staining. Cytoplasmic staining and tumor-associated immune cells, such as macrophages, were excluded from scoring. The tumor proportion score (TPS) was calculated as follows:

TPS (%) = (PD-L1–positive tumor cells / Total number of tumor cells) × 100. (https://www.accessdata.fda.gov/cdrh_docs/pdf16/p160046c.pdf).

**Figure 2:**
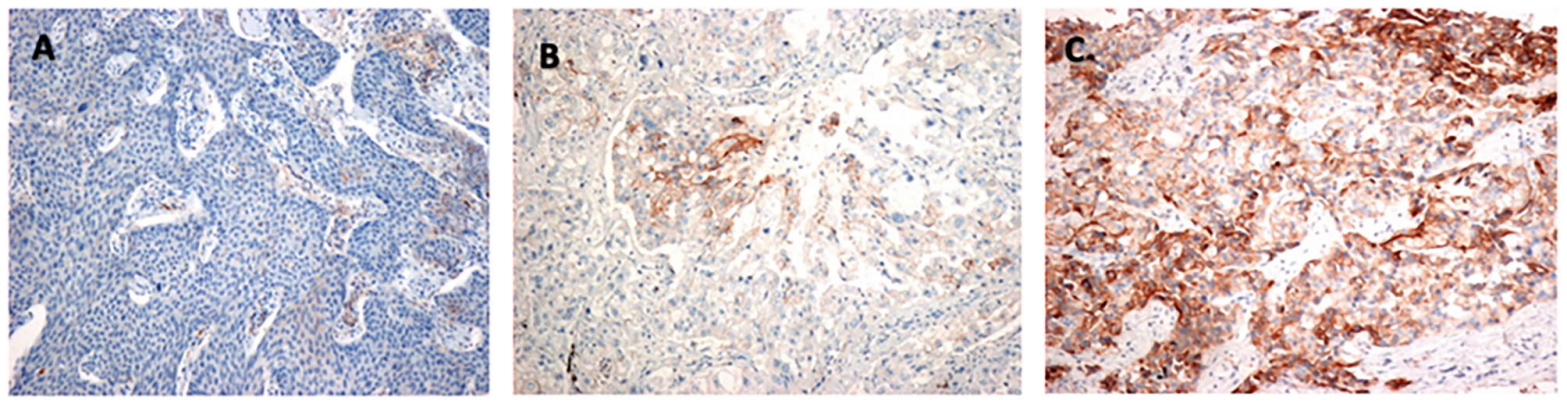
Identification of PD-L1–positive tumor cells: **(A)** Negative staining (TPS < 1%). **(B)** Weakly positive staining (TPS 1–49%). **(C)** Highly positive staining (TPS ≥ 50%). PD-L1, programmed death-ligand 1; TPS, tumor proportion score.

The TPS was used to categorize PD-L1 expression status as follows: <1% (negative staining), 1%−49% (weakly positive staining), and ≥50% (highly positive staining). All tumors with TPS ≥1% were considered PD-L1–positive. Figure 2 shows representative images of the three PD-L1 categories.

### Statistical Analysis

Statistical analyses were performed using SPSS, version 20.0 (SPSS Inc., Chicago, IL, USA). Data are presented as the number and frequency or the mean ± standard deviation (SD). The association between two continuous variables was analyzed by the Mann–Whitney U test or *t*-test. The association between two categorical variables was evaluated by the Chi-square test. Significance was established at *p* < 0.05.

## Results

### Patient Population

The general characteristics of the study population are shown in Table 1. The average age was 62.23 ± 9.51 years, and 64% of patients were younger than 65 years. Men represented 67.4% of the study population. Smoking was reported by 44.2% of patients. *EGFR* mutations were detected in 36% of the study population. PD-L1 expression was negative in 40.7% of cases and positive in 59.3% of cases, including 32.6% with weak PD-L1 expression (1–49% of cells) and 26.7% with strong PD-L1 expression (≥50% of cells). The mean SUV_max_ was 11.09 ± 3.94. Other characteristics are summarized in Table 1.

**Table 1:** General patient characteristics (*n* = 86).

**Characteristics**		**n**	**Percentage (%)**
Sex	Male	58	67.4
	Female	28	32.6
Age, years	<65	55	64.0
	>65	31	36.0
Smoking	Yes	38	44.2
	No	48	55.8
*EGFR* mutation	No	55	64.0
	Yes	31	36.0
PD-L1 expression level	Negative	35	40.7
	1−49%	28	32.6
	≥50%	23	26.7
SUV_max_	Mean ± SD	11.09 ± 3.94	
Survival	Yes	78	90.7
	No	8	9.3
T stage	T1	9	10.5
	T2	9	10.5
	T3	29	33.7
	T4	39	45.3
N stage	N0	5	5.8
	N1	3	3.5
	N2	33	38.4
	N3	45	52.3
Pleural metastasis	Yes	34	39.5
	No	52	60.5
Lymph metastasis	Yes	16	18.6
	No	70	81.4
Brain metastasis	Yes	7	8.1
	No	79	91.9
Liver metastasis	Yes	9	10.5
	No	77	89.5
Adrenal metastasis	Yes	7	8.1
		79	91.9
Bone metastasis	Yes	43	50.0
	No	43	50.0
Other lung metastasis	Yes	39	45.3
	No	47	54.7

*EGFR, epidermal growth factor receptor; PD-L1, programmed death-ligand 1; SUV_max_, maximum standard uptake value; T, tumor; N, node; SD, standard deviation*.

**Figure 3:**
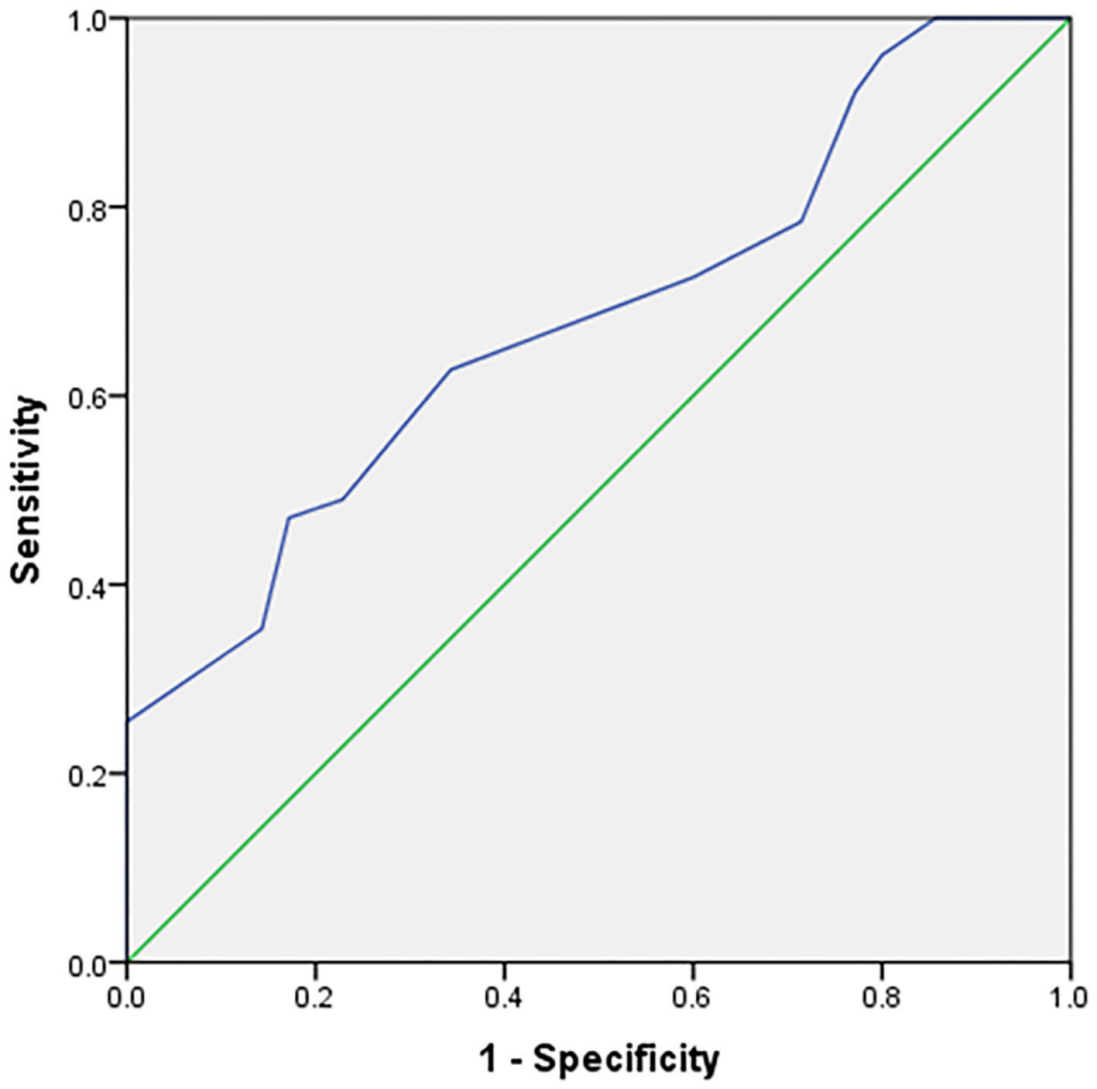
The ability of SUV_max_ to predict PD-L1 expression.

The expression of PD-L1 was evaluated in 86 tumor samples by immunohistochemical analysis. A positive association was identified between SUV_max_ from ^18^F-FDG PET/CT imaging and PD-L1 expression. SUV_max_ was significantly higher in PD-L1–positive tumors than in PD-L1–negative tumors (12.24 ± 4.01 vs. 9.43 ± 3.22, respectively; *p* = 0.001). The ability of SUV_max_ to predict PD-L1 expression was determined (Figure 3) by performing receiver operating characteristic curve analysis, which showed revealed an area under the curve (AUC) of 0.681 (95% confidence interval [95% CI] = 0.570–0.793, *p* = 0.004).

**Figure 4:**
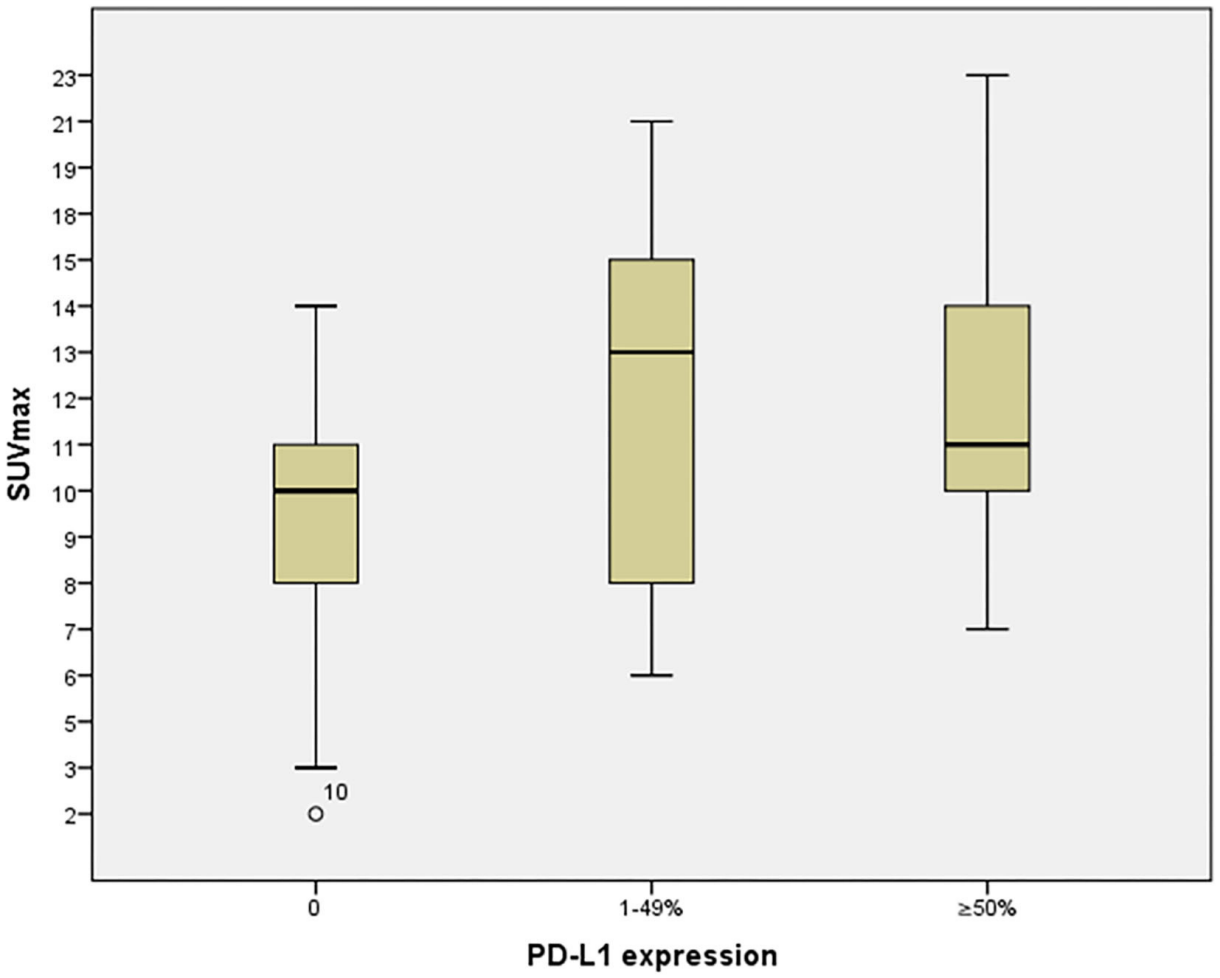
The relationships between SUV_max_ and PD-L1 expression.

Table 2 shows the relationship between SUV_max_ and PD-L1 expression. Significance differences were identified between SUV_max_ in PD-L1–negative and SUV_max_ for all PD-L1–positive cases (*p* = 0.001), weakly PD-L1–positive cases (1–49%, *p* = 0.005), and strongly PD-L1–positive cases (≥50%, *p* = 0.003). These relationships are displayed graphically in Figure 4.

**Table 2:** The association between SUV_max_ and PD-L1 expression.

	**n**	**Mean ±SD**	***P*-value**
SUV_max_ in PD-L1–negative	35	9.43 ± 3.22	0.005 (^*^)
SUV_max_ in PD-L1 positive (1–49%)	28	12.18 ± 4.23	
SUV_max_ in PD-L1 negative	35	9.43 ± 3.22	0.003 (^*^)
SUV_max_ in PD-L1 positive (≥50%)	23	12.30 ± 3.81	
SUV_max_ in PD-L1 positive (1%−49%)	28	12.18 ± 4.23	0.913
SUV_max_ in PD-L1 positive (≥50%)	23	12.30 ± 3.81	
SUV_max_ in PD-L1 negative	35	9.43 ± 3.22	0.001 (^*^)
SUV_max_ in PD-L1 positive (total)	51	12.24 ± 4.01	

(^*^) Significant according to t-test.

*SUV_max_, maximum standard uptake value; PD-L1, programmed death-ligand 1; SD, standard deviation*.

Table 3 shows all the associations between patient characteristics and PD-L1 expression status. PD-L1 expression was significantly associated with SUV_max_ (*p* = 0.001), tumor size (*p* = 0.022), and *EGFR* mutation status. However, we found no associations with age, sex, carcinoembryonic antigen (CEA) level, cytokeratin 19 fragment (CYFRA 21-1) level, smoking, survival status, T stage, N stage, or any metastasis location (pleural, lymph, brain, liver, adrenal, bone, or other lung).

**Table 3:** Comparisons between patient characteristics and PD-L1 expression.

**Characteristics**		**PD-L1–Positive**	**PD-L1–Negative**	**p-value**
Age, years (mean ± SD)		61.67 ± 9.42	63.06 ± 9.73	0.509
CEA level		60.94 ± 108.23	88.40 ± 209.04	0.415
CYRFRA 21-1 level		10.38 ± 14.82	9.95 ± 12.43	0.898
Size of tumor		4.98 ± 2.26	3.91 ± 1.77	0.022 (^*^)
SUV_max_		12.24 ± 4.01	9,43 ± 3.29	0.001 (^*^)
Sex	Male	35	23	0.777
	Female	16	12	
Age, years	<65	34	21	0.527
	>65	17	14	
Smoking	Yes	20	18	0.263
	No	31	17	
Survival	No	5	3	0.847
	Yes	46	32	
T stage	T1	4	5	0.550
	T2	4	5	
	T3	18	11	
	T4	25	14	
N stage	0	4	1	0.286
	1	3	0	
	2	17	16	
	3	27	18	
*EGFR* mutation	Negative	37	18	0.045 (^**^)
	Positive	14	17	
Pleural metastasis	Yes	18	16	0.332
	No	33	19	
Lymph metastasis	Yes	12	4	0.157
	No	39	31	
Brain metastasis	Yes	5	2	0.496
	No	46	33	
Liver metastasis	Yes	6	3	0.635
	No	45	32	
Adrenal metastasis	Yes	3	4	0.355
	No	48	31	
Bone metastasis	Yes	26	17	0.826
	No	25	18	
Other lung metastasis	Yes	25	14	0.426
	No	25	20	

(^*^) Significance determined by t-test.

(^**^) Significance determined by Chi-square test.

*PD-L1, programmed death-ligand 1; CEA, carcinoembryonic antigen; CYFRA 21-1, cytokeratin 19 fragment; SUVmax, maximum standard uptake value; T, tumor; N, node; EGFR, epidermal growth factor receptor; SD, standard deviation*.

## Discussion

^18^F-FDG PET/CT can reveal disease at the molecular level prior to the occurrence of anatomical structural alterations, detected via observing changes in metabolism. SUV_max_ measured by ^18^F-FDG PET/CT has excellent reproducibility and is widely available; therefore, this measurement is often used to establish precise diagnoses, perform TNM staging, plan radiation therapy, and monitor therapeutic effects for lung cancers in comparison with other imaging modalities such as computed tomography, magnetic resonance imaging, and scintigraphy (10–12).

Takada et al. demonstrated that the metabolic features of lung cancers expressing PD-L1 on ^18^F-FDG PET/CT were associated with other parameters, such as smoking status, pleural invasion, and SUV_max_ (13). In our study, we discovered no relationships between PD-L1 and smoking status, pleural invasion, or any other invasion type.

In our investigation, ^18^F-FDG PET/CT measurements were able to predict PD-L1 expression status in stage IV adenocarcinoma lung cancer patients, with an AUC of 0.681. This outcome differs slightly from results reported by Cui Y et al., who studied 73 patients with adenocarcinoma lung cancer and found an AUC of 0.855 for the prediction of PD-L1 expression using SUV_max_ (14). This difference could be due to differences in the patient populations between these two studies.

SUV_max_ has been found to be a prognostic indicator for both early and advanced NSCLC (15). A meta-analysis revealed that a high SUV_max_ is associated with poor OS in patients with NSCLC (16). Although we obtained survival data from the stage IV patients in our study, the OS rate is still being evaluated. Preoperative SUV_max_ at the primary lesion is a more accurate indicator of nodal metastases when a cutoff of 3 is used (17). Almost all patients in our study had an SUV_max_ > 3 because they were all in stage IV with metastases. Increased PD-L1 expression is associated with worse prognosis in patients with NSCLC (18), supporting the concept that enhanced PD-L1 expression in tumor cells facilitates the evasion of host immune monitoring, promoting disease progression (19). However, Kerr et al. demonstrated that increased PD-L1 expression was associated with better OS in patients with resected NSCLC (20). Thus, high PD-L1 expression has been associated with both favorable and adverse prognoses (20). PD-L1 expression was associated with poorer OS prognosis in a study examining the relationships between PD-L1 expression and various clinicopathologic factors in 90 resected NSCLC patients, including various adenocarcinoma subtypes (21). The preoperative SUV_max_ at the primary lesion measured during ^18^F-FDG PET/CT is a more efficient index of nodal metastasis than tumor size, and SUV_max_ can predict regional lymph node metastases (22).

In patients with early-stage lung cancer who are suitable for resection, preoperative SUV_max_ is associated with PD-L1 expression in NSCLC patients (22), as demonstrated in another study (13). An SUV_max_ of 8.6 is associated with PD-L1 expression (TPS 11%) and is an independent prognostic factor for OS in lung squamous cell carcinoma (23). Additionally, elevated PD-L1 expression and a high SUV_max_ (>11.2) are both independently associated with poor OS in surgical lung squamous cell carcinoma (24). A significant difference in OS was identified between individuals with lung adenocarcinoma with SUV_max_ of 2.9 (25).

Although clinical research studies examining the association between SUV_max_ and PD-L1 are limited, the findings remain controversial. Determining the relationship between PD-L1 expression and SUV_max_ can determine optimal treatment selection. Previous clinical trials have demonstrated that the expression of immune checkpoints, such as PD-L1, in various patient populations can predict treatment efficacy, including pembrolizumab vs. chemotherapy, pembrolizumab vs. platinum-based chemotherapy for advanced NSCLC (26–28). SUV_max_ is considerably higher in patients with positive PD-L1 expression than in those with negative PD-L1 expression (13). This finding suggests that combining the evaluation of PD-L1 expression and SUV_max_ in the primary tumor may help predict stage IV adenocarcinoma lung cancer prognosis.

Immuno-PET imaging may become a routine clinical assessment tool in this field in the near future. By defining tumors using TKI-PET and immuno-PET, we can tailor NSCLC therapy (29). Whole-body PD-L1 PET can also be conducted on NSCLC (30). We could obtain more detailed information on PD-L1 expression by using immune-PET because immune-PET delays the resolution of unresolved issues. Immune-PET can provide more precise information regarding PD-L1 expression while also consistently collecting the SUV_max_ of the primary site. This study may gain increased significance in the future as this imaging method becomes more regularly used.

Several limitations existed in this study. First, this is a cross-sectional study, which did not allow us to evaluate the response to treatment PD-L1–targeted treatment. Additional studies with larger, externally validated cohorts remain needed to elucidate the value of PD-L1 and SUV_max_ for evaluating treatment and prognosis. Second, glutamine transporters (GLUT1) and hexokinase II should be included in future investigations. Further studies are also essential to evaluate the value of FDG PET/CT in predicting immunotherapy response.

## Conclusion

The SUV measured in the primary lesion was valuable for predicting PD-L1 expression status in stage IV adenocarcinoma lung cancer patients. Therefore, SUV_max_ may play a role in predicting the efficacy of PD-L1 immunotherapy in patients with stage IV lung adenocarcinoma.

## Data Availability Statement

The original contributions presented in the study are included in the article/supplementary material, further inquiries can be directed to the corresponding authors.

## Ethics Statement

This study was approved by the Ethics Committee of Hanoi Medical University (accession no. NCS02/HMU-IRB). The patients/participants provided their written informed consent to participate in this study.

## Author Contributions

BTC and PCP gave a substantial contribution in acquisition, analysis, and data interpretation. BTC, P-VT, and NMD prepared, drafted, and revised manuscript critically for important intellectual content. All authors gave the final approval of the version to be published and agreed to be accountable for all aspects of the work, ensuring that questions related to the accuracy or integrity of any part of the work are appropriately investigated and resolved.

## Conflict of Interest

The authors declare that the research was conducted in the absence of any commercial or financial relationships that could be construed as a potential conflict of interest.

## Publisher's Note

All claims expressed in this article are solely those of the authors and do not necessarily represent those of their affiliated organizations, or those of the publisher, the editors and the reviewers. Any product that may be evaluated in this article, or claim that may be made by its manufacturer, is not guaranteed or endorsed by the publisher.
